# Three years of weekly DEMs, aerial orthomosaics and surveyed shoreline positions at Waikīkī Beach, Hawai‘i

**DOI:** 10.1038/s41597-024-03160-z

**Published:** 2024-03-29

**Authors:** Anna B. Mikkelsen, Kristian K. McDonald, Julianne Kalksma, Zachary H. Tyrrell, Charles H. Fletcher

**Affiliations:** 1 Department of Earth Sciences, School of Ocean and Earth Science and Technology, University of Hawai‘i at Mānoa, Honolulu, HI 96822 USA; 2 Department of Ocean and Resources Engineering, School of Ocean and Earth Science and Technology, University of Hawai‘i at Mānoa, Honolulu, HI 96822 USA

**Keywords:** Environmental impact, Geography, Climate and Earth system modelling

## Abstract

In this dataset, we present 128 coastal surveys conducted between 2018 and 2021 at Kahaloa Beach, also known as the Royal Hawaiian Beach, in Waikīkī, Hawai‘i. Surveys were conducted on a near-weekly basis, providing a 0.5 m digital elevation model, an orthorectified image mosaic with 0.03 m resolution, and shoreline vectors at MHHW and MSL, along with a surveyed shoreline position for each survey. We captured overlapping images using a small Unoccupied Aerial System (sUAS), processing the imagery with photogrammetric software to produce orthomosaics and Digital Terrain Models (DTM). Simultaneously, the shoreline position and reference points for sUAS-derived products were surveyed using total station and rod-mounted surveying prism. A quality assessment of 424 randomly sampled points across two surveys showed normally distributed errors of DTM elevations (µ_1_ = 0.0060 m; *σ*_1_ = 0.0998 m; µ_2_ = 0.0035 m; *σ*_2_ = 0.0680). Elevation uncertainties were quantified as 95% confidence intervals (±0.0130 m and ±0.0095 m). These data are intended to encourage research on reef-fringed beaches and provide a dataset for evaluating the accuracy of satellite-derived shorelines at reef-fringed beaches.

## Background & Summary

Accurate shoreline monitoring is imperative to understand coastal risks, quantify both long-term and short-term change rates, and provide essential information to coastal managers worldwide^1–4^. With an estimated 896 million people currently residing in low-lying coastal areas, a figure projected to reach 1 billion by 2050^5^, safeguarding coastal environments is critical. Sandy beaches and dunes make up about 30% of the global coastline^6^ and are dynamic systems that respond to environmental factors such as waves and sea level changes^7^. These sandy beaches hold cultural significance for many island and coastal communities and often contribute significantly to their economy through coastal tourism^8^. Furthermore, they function as critical storm buffers^9^ and provide essential habitats for various species, including monk seals and turtles^10^. However, with impacts of rising sea levels and intensifying storminess relating to climate change^11^, combined with increased coastal development, leading to the coastal squeeze of shoreline, many of these sandy environments are suffering from erosion or landward shoreline migration^6,12,13^. With sea levels expected to rise for centuries to come^14^, the threat to coastal land loss intensifies. Therefore, acquiring comprehensive data to understand coastal responses is essential for effective coastal zone management.

Conducting consistent *in-situ* surveys is challenging because of requirements for time, personnel, and equipment. Despite their integral role in enhancing the understanding of coastal morphodynamics, only a limited number of long-term, consistent coastal study datasets exist^15–19^. In recent years, Satellite-Derived Shoreline (SDS) data have emerged as a crucial tool for monitoring coastlines^20–22^. Here, shorelines are delineated using publicly available satellite imagery, creating a time series of shoreline positions from which various metrics, such as long-term erosion/accretion rates and seasonal variability^23^, and basin-scale patterns^24^ can be derived. Additionally, they can be used for future shoreline projections^21,22^. As this method becomes increasingly prevalent in coastal monitoring, it is imperative to benchmark its reliability and accuracy with ground truth survey data. Yet, there are only few datasets encompassing long-term consistent *in-situ* monitoring. Among them are datasets from 1) Duck beach, USA^15^, 2) Narrabeen, Australia^16,17^, 3) Truc Vert, France^25^, and 4) and Torrey Pines, USA^26^. These four datasets are currently used in standardized benchmarking efforts^27^ and predominantly feature large continental, low-sloping silicate beaches. However, previous research has indicated variations in the accuracy of satellite benchmarking across different sites^23^. Given that no thorough dataset exists to inform accuracy on reef-fringed environments to our knowledge, we aim to partially fill this gap by providing 3.5 years of benchmarking data for a carbonate reef-fringed beach. This dataset comprises near-weekly surveys from 2018 to 2021 of Kahaloa Beach, Hawai‘i. For each survey, we employed small Unoccupied Aerial Systems (sUAS) to collect aerial imagery, which was processed using photogrammetry software to create digital terrain models (DTMs) with 0.5 m resolution and orthorectified image mosaics with 0.03-0.04 m resolution. Ground surveys, conducted with a survey-grade total station, were used to establish shoreline position at the mean lower low water mark (MLLW), and capture accurate reference points for the photogrammetry processing. Additionally, beach volumes were derived from the DTM. Each of these products are described in detail in *Methods* below.

This data collection was initially motivated to respond to chronic erosion concerns at the central Kahaloa Beach in Waikīkī. Previously, analysis from eight and twenty-two months of data collection have been published to understand the drivers of beach change and beach responses to hurricanes^28,29^. This accounts for 56% of the total data collected (72 surveys), which are accessible in a Mendeley data repository^30^. In this report, we present an additional 18 months of data (56 surveys), extending the time series to 40 months^31^. Additionally, individual shoreline vectors at Mean Sea Level (MSL) and Mean Higher High Water (MHHW) contours are provided for ease of reuse in satellite shoreline benchmarking efforts. By consolidating these data in one data descriptor and platform, we provide a valuable asset to enhance the understanding of complex reef-fringed beaches. Additionally, these data serve as a critical benchmarking dataset for evaluating satellite derived shorelines in reef-fringed beach environments.

## Site Description

**Fig. 1:**
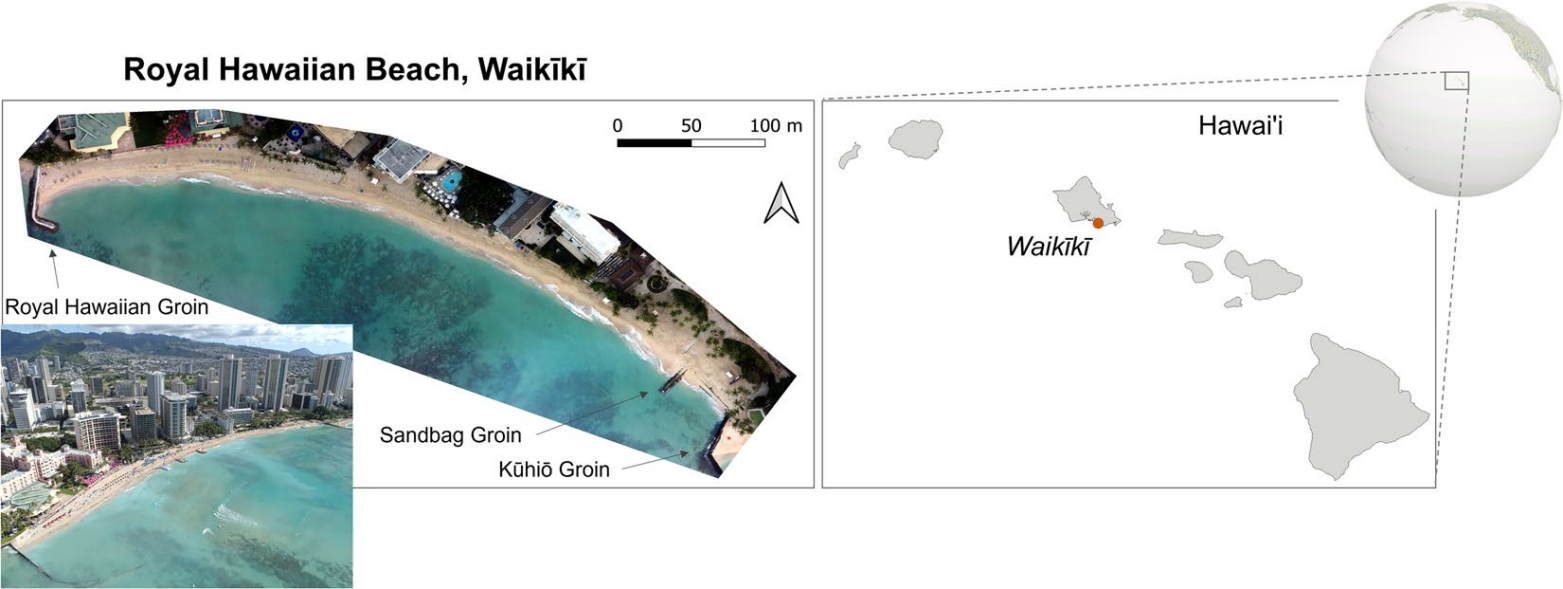
Kahaloa Beach in Waikīkī. The study site is a narrow reef-fronted beach. Two terminal groins bound the beach, and an additional stub groin was placed to retain sand in the easternmost portion of the beach.

Kahaloa Beach, commonly referred to as The Royal Hawaiian Beach, is a popular beach located in the heart of Waikīkī (Fig. 1). It is a crescent-shaped carbonate beach, fronted by a fringing reef. It extends 520 m and is bounded by two terminal shore-normal groins that limit the alongshore sediment exchange into and out of the littoral cell^32^. Large sediment gains or losses are facilitated primarily through cross-shore transport via a subtle sand channel located in the center of the nearshore region^29^. Longshore sediment exchange has been shown as a mechanism for re-distribution of sediment within the littoral cell^28,32^.

The beach is characterized by chronic erosion, necessitating intervention to maintain usable beach width. Starting in 1890’s, the beach has seen modifications in the form of encroachment of development, beach renourishment, structural change such as groins and seawalls, which have been constructed and removed through the last century, and dredging of the coral reef fronting parts of this beach^33^.

The chronic erosion is typically punctuated by seasonal morphologic change where summer south swell corresponds to accretion and local trade-wind swell (intermittent year-round) promote erosion^29,32,34^. The nearshore comprises a gently sloping fringing coral reef a few hundred meters wide^33^. In the immediate nearshore, a sand field with intermittent rocky substrate (fossilized reef) extends seaward from the toe of Kahaloa Beach approximately 100–200 m. Foreshore slopes at Kahaloa Beach have been measured to average 0.149 from the Low Water Mark (LWM) to the first beach berm (not shown).

Sand grain size varies due to numerous engineering and re-nourishment projects. The offshore sand source used for two renourishment projects, one in 2012 and another in 2021, had a median diameter of 0.3 mm, matching the existing beach sand in distribution, texture, and color. Notably, Kūhiō Beach Park (the easternmost section) exhibited coarser sand (D50 = 0.80 mm) compared to the rest of the beach, where grain sizes ranged from 0.29 mm to 0.40 mm^35^. The dredged sand primarily consists of carbonate material, predominantly composed of calcareous skeletal fragments from marine organisms such as corals, coralline algae, mollusks, echinoids, and foraminifera, with minimal coarse material or fines.

The Hawaiian Islands are characterized by a relatively low tidal range, which reaches a maximum of 1 m during the summer spring perigee. The prevailing wave conditions in Hawai‘i are closely tied to the seasons^36,37^. Waikīkī experiences two primary wave fields: 1) Summer swells, prominent between April and October, originating in the Southern Ocean with periods spanning 14 to 22 s and heights ranging from 1 to 5 m; and 2) Locally generated tradewind waves, characterized by shorter periods (6–10 s) and relatively lower heights (1–3 m), persisting throughout the year but becoming more frequent in the summer. Winter swells generated by North Pacific storms generally have a minimal impact on Waikīkī beaches, as they are sheltered by the island. Conversely, swells associated with Kona Storms (typically in winter) or Tropical cyclone swells (typically occurring from June to November), can introduce substantial wave energy and drive significant coastal change^38,39^, including beach accretion^29^.

During the survey period, three notable changes were made to Kahaloa Beach (Fig. 1): (1) In November 2019, a small 30 m stub groin constructed from large sandbags was installed to retain sand in the easternmost section of the beach. (2) In May 2020, a replacement of the Royal Hawaiian Groin was built at the westernmost end of the beach, shorter but denser, to replace a failing groin that had been in place since 1930^33^. (3) In April through May 2021, a beach renourishment project was undertaken, involving the pumping of sand from offshore deposits into a drying basin, after which it was trucked onto Kahaloa Beach over a 2–3-week period. This brought in approximately 15,291 m^3^; widening the beach by 9.1 m on average and increasing the dry sandy area by 4,831 m^2^ (Figs. 1 and 4)^40,41^. A prior beach nourishment took place in 2012^32^ to retain usable beach width and it is projected that sand nourishment will be required every 5–10 years to maintain the beach.

**Fig. 2:**
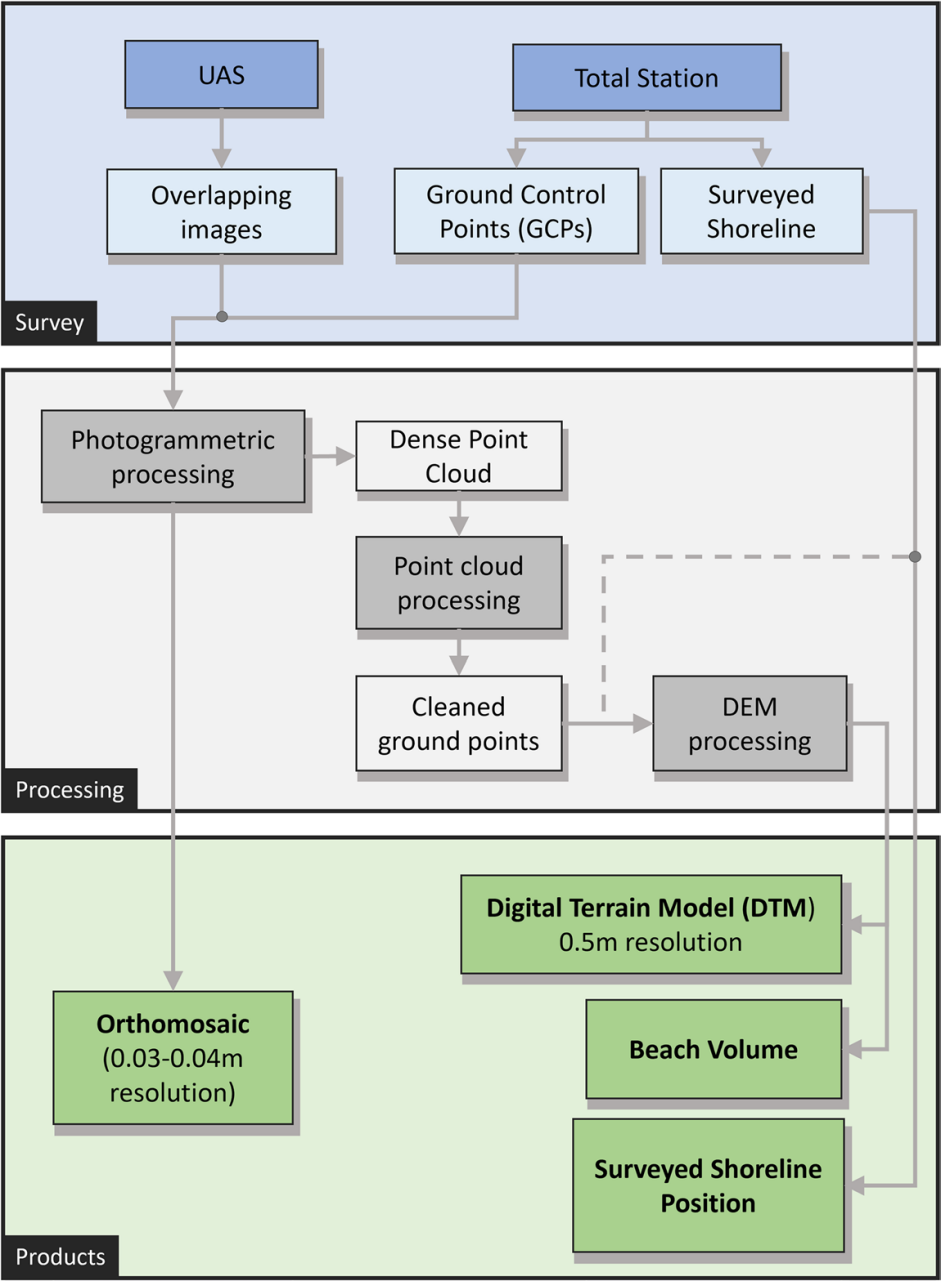
Flowchart of methodology. Following surveys using sUAS and total station, data is processed with photogrammetric software, after which the point cloud is processed, and resulting outputs include a surveyed shoreline, orthomosaic, Digital Terrain Model and beach volume.

**Fig. 3:**
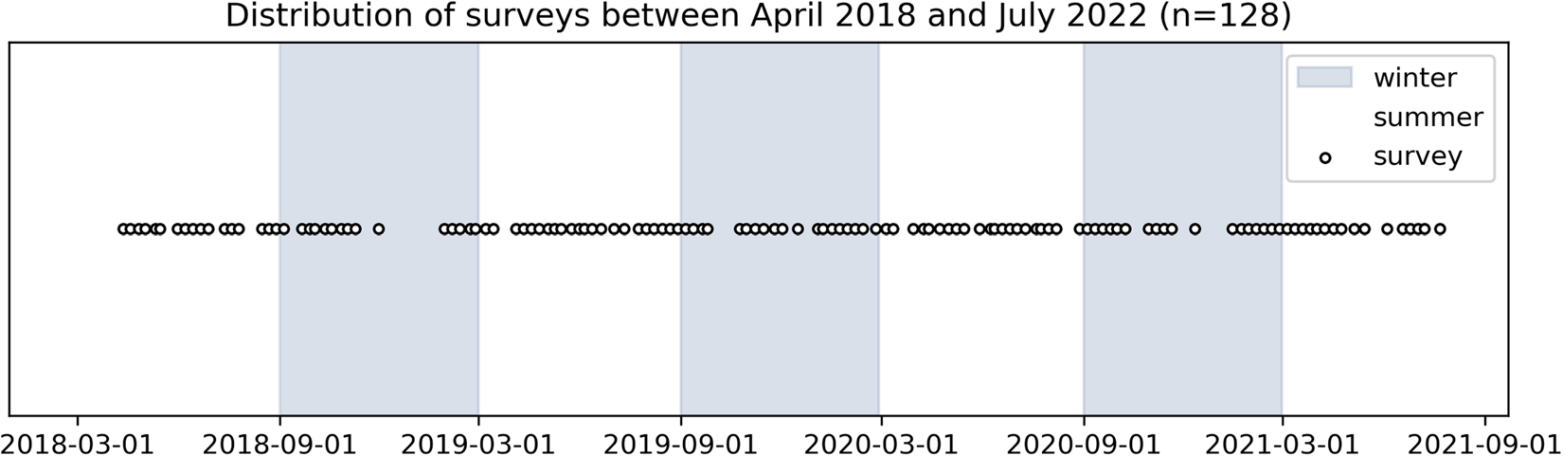
Survey frequency. Surveys were conducted near-weekly, with some gaps due to beach construction projects or weather conditions.

**Fig. 4:**
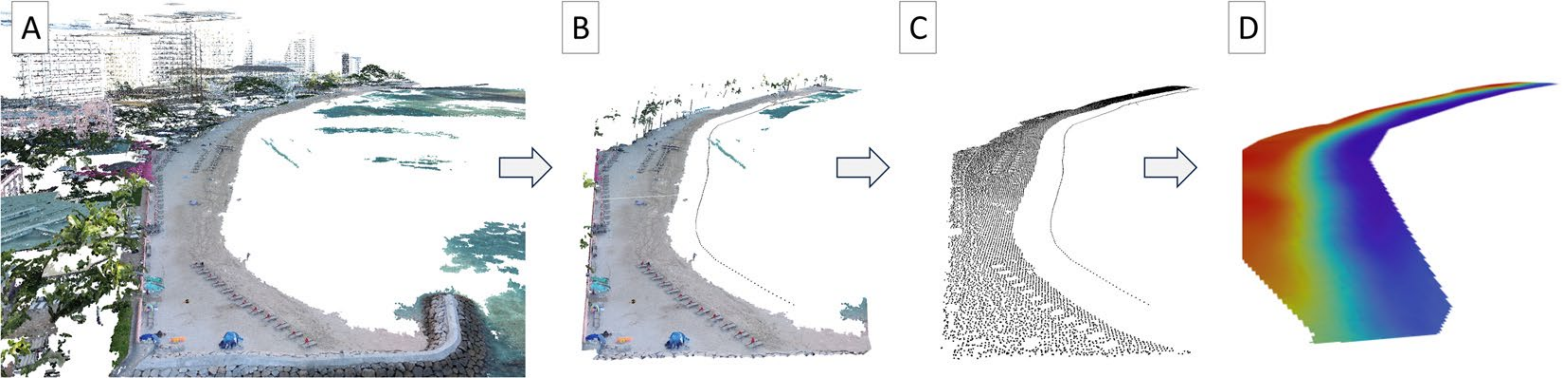
Point cloud processing to identify ground points and remove outliers. The dense point cloud exported from Agisoft Metashape (**A**) is clipped (**B**) and processed to remove all non-ground points, as well as points below 0.6 m elevation (**C**). The ground points are merged with the surveyed LWM (shoreline points in panels B & C), and interpolated into a 0.5 m resolution DTM (**D**).

## Methods

### Field work

We conducted 128 near-weekly ground and aerial beach surveys at Kahaloa Beach, Waikīkī, from April 2018 to July 2021 (Fig. 3). These surveys were initially motivated to better understand beach response to both seasonal and tropical-storm-generated waves. A consumer-grade sUAS (DJI phantom4 Pro v1 & v2) and the flight planning software package DroneDeploy was used to collect overlapping images at 120 m altitude, resulting in a ground sampling distance of 3-4 cm. All images were taken at nadir with 80% side-lap/end-lap. The sUAS imagery was georeferenced using ground control points (GCPs) described below; RTK or onboard sUAS GPS was not used (Fig. 2).

Camera settings varied depending on light conditions, but high shutter speeds (1/1000–1/600) were prioritized to reduce motion blur. Each survey was conducted in the morning around sunrise to capture the beach prior to crowds and objects (e.g., beach umbrellas, chairs, tents, etc.), irrespective of tide level and ocean conditions. Repeat surveys were planned weekly; however, given the weather dependent nature of sUAS flights, surveys were conducted around optimal weather (low winds, no rain) for each week resulting in revisit times between 5 and 15 days (Fig. 3).

Seven 1 × 1 m vinyl targets functioned as GCPs and were spaced equidistant about 100 m alongshore for each survey. The position of GCPs were surveyed using a rod-mounted prism and a Leica TS16 Robotic Total Station with millimeter-level accuracy. Additionally, points were collected every 5–15 m along the seaward edge of the foreshore, here marked by the position of the top of the beach toe, also approximately equal to the low water mark (LWM)^39,42^. Existing benchmarks provided a spatial reference using the WGS 1984 UTM Zone 4 projection. Elevations were measured with respect to local mean sea level (LMSL; Datums – NOAA Tides and Currents, present epoch: 1983–2001).

### Data processing

Following standardized USGS protocols^43,44^ for photogrammetry processing, Agisoft Metashape (formerly Photoscan) was used to generate a dense point cloud and orthomosaic (Fig. 2). An initial sparse point cloud was created with GCPs manually identified and subsequently verified for each image containing a GCP. After importing the surveyed GCP coordinates and re-aligning the sparse cloud, an iterative error reduction process was initiated, in which points above a certain threshold were removed and the sparse point cloud re-aligned. This iterative process was repeated until uncertainty values were below a specific threshold. Approximately 10% of the points were removed at each iteration, initially reducing the *“Reconstruction Uncertainty”* to 10, followed by reducing “*Projection Accuracy”* to 3, and lastly reducing *“Reprojection Error”* once, by eliminating the 10% of the point cloud with highest values. From here, a dense point cloud, DEM, and orthomosaic was generated from Agisoft Metashape.

The dense point cloud was post-processed using rapidlasso LAStools to eliminate data artifacts such as beach umbrellas and canoes, identify ground points, and remove outliers. The LAZ point cloud was initially cropped to exclude landward and ocean points beyond the study area. It was then tiled, sorted, thinned, and removed of erroneous points. In order to minimize the impact of the moving water interface and wave runup, we removed all points located in the run-up zone. This involved eliminating all points situated below an elevation threshold of 0.6 meters above mean sea level. This threshold was determined empirically, considering high errors and sporadic points resulting from saturated sand and wave run-up in the swash zone, which affect the overall quality of the DTM. The post-processed point clouds were manually assessed, and any remaining points deemed erroneous were removed, if necessary. We then merged the remaining point cloud with the surveyed shoreline points of the LWM, approximately −0.251 m relative to MSL. A surface was interpolated across all points at 0.5 m resolution using natural neighbor interpolation, then smoothed using mean cell values within a 5 m radius circular to eliminate any remaining elevation anomalies caused by beach artifacts. The 5 m radius was selected empirically to balance effective smoothing without masking weekly changes or introducing errors compared to ground-sampled elevations (see Technical Validation).

**Fig. 5:**
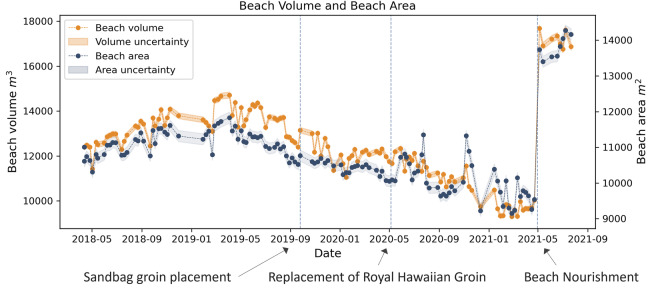
Beach volume (orange) and beach area (blue) throughout the survey period. The vertical lines denote beach management interventions, the most recent being the beach nourishment that occurred in May 2021. Shadings Represent uncertainty bands for volume and area calculations.

Beach volume (Fig. 5) was calculated in ArcGIS pro using the *Surface Volume* tool. Here, the floor was defined by the MHHW equivalent to 0.329 m above MSL. As a result, the seaward boundary was the intersection of the DTM surface with the MHHW contour, while the landward extent was constant and primarily determined by walkways and buildings.

MSL and MHHW contours were generated using the *contour* tool in ArcGIS and subsequently reviewed for necessary edits. Adjustments were made if a contour was interrupted by the Kūhiō shore normal groin or if contours inaccurately extended seaward at the beach ends. In these cases, the contour was completed by digitizing the shoreline feature in the image mosaic. This would typically occur if the beach was eroded, resulting in the DTM encompassing a larger area than the data collection zone, occasionally resulting in sporadic offshore discrepancies.

## Data Records

The data are available at figshare^31^. This dataset consists of five (5) products derived from each field survey. The (1) Digital Terrain Model (DTM) and (2) orthomosaic are provided in LZM compressed tagged image file format (TIFF) in WGS 1984 UTM Zone 4 projection (EPSG:32604). The DTMs have a resolution of 0.5 m, and a vertical uncertainty quantified as the 95% confidence interval around the mean (±0.0113 m; see *Technical Validation* below). Elevations are measured with respect to local mean sea level (LMSL) (NOAA tides and currents; station ID 1612340). The orthomosaics are produced from each survey to create a mosaic without distortion. The resolution varies slightly between surveys and is 3-4 cm.

Surveyed shoreline points (3) marking the seaward edge of the foreshore are provided in a.txt file, with easting, northing, and elevation values, respectively, as columns and each row representing a surveyed point. These are also in WGS 1984 UTM Zone 4 projection and elevations are with respect to LMSL. The Leica TS16 Robotic Total Station, which was used to sample these data points, provides sub-centimeter accuracy. The primary uncertainty of this measurement is therefore inconsistencies in identifying the beach toe, and wave impacts that could potentially shift the position of the rod-mounted prism at the time of measurement (see *Technical Validation* below). For effective comparison with satellite-derived shorelines, shoreline contours (4) are provided for MHHW and MSL for each survey. Additionally, a contour produced from the surveyed shorelines points representing the LWM is included. These are provided as individual geojson files, named by YYYYMMDD_datum.geojson, and each file contains two attributes: the contour elevation relative to MSL (0 m) and the date of the survey. Lastly, a text file (5) containing calculated metrics for each survey is included. Each row represents a survey labeled with the survey date (YYYYMMDD), and metrics include beach volume, 2D, and 3D area calculated from the DTM (Fig. 5), as well as volume ± elevation uncertainty times the 3D area.

## Technical Validation

### Uncertainty in sUAS-derived DEM

**Fig. 6:**
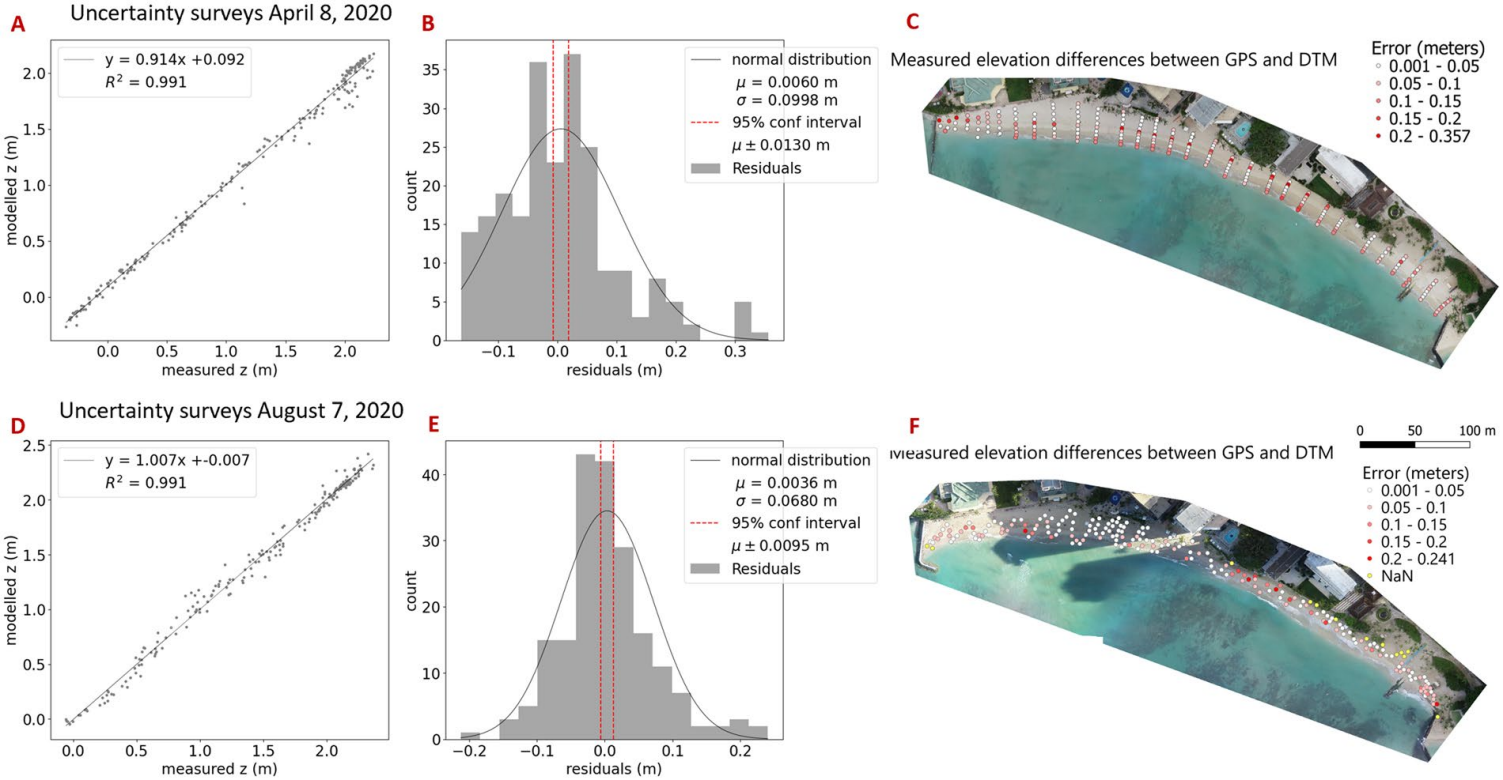
Elevation uncertainty of sUAS derived DEM. This figure shows the results of two separate uncertainty surveys conducted on April 8, 2020 (panels **A**–**C**) and August 7, 2020 (panels **D**–**F**). A 1:1 plot of measured (x-axis) and modeled (y-axis) elevation values are shown in panels **A** (n = 228) and **D** (n = 196). Residuals are represented in a histogram in panels **B** and **E**. The 95% confidence interval (red vertical bands) was calculated as 1.96 times the standard error (SE). Panels **C** and **F** show the distribution of points across Kahaloa Beach, colored by elevation difference between measured and modeled elevations, with white being lowest (0.001–0.05 m) and red being highest (0.20–0.357 m). Yellow points were collected with our total station but did not overlap with the derived DEM.

To assess the quality in the elevation values of the sUAS-derived DEMs, two separate surveys were conducted in which measured point elevations (collected with the Leica TS16 Robotic Total Station, n = 228 and n = 198) were compared to modeled point elevations from the corresponding sUAS-derived DEM. Figure 6 shows the distribution of these points collected across the beach and their comparison with the measured elevation values for surveys conducted on April 8, 2020 (Fig. 6A–C) and August 7, 2020 (Fig. 6A–F). Panels a and d display a one-to-one comparison between the measured and modeled elevation values, while panels b and e present the histogram of residuals. Panels c and f illustrate the distribution of points colored by their associated errors. Essential statistical parameters, such as the mean (0.0060 m and 0.0035 m), standard deviation (0.0998 m and 0.0680 m), root mean square error (RMSE; 0.0998 m and 0.0680 m), and standard error (0.0066 m and 0.0049 m), were computed, assuming a normal distribution.

To quantify uncertainties in the elevation values, 95% confidence intervals were calculated as 1.96 times the standard error (0.0130 m and 0.0095 m). Subsequently, uncertainties in beach volume calculations were estimated by multiplying the 95 percent confidence interval with the 3D beach area. Uncertainties in surveyed shoreline positions are attributed to uncertainty or inconsistencies in locating the LWM feature at ±0.3 m in the cross-shore direction.

## Usage Notes

The purpose of sharing these data serves a dual role: 1) To encourage research focused on reef-fringed beaches, and 2) to offer a dataset for evaluating the accuracy of satellite-derived shoreline (SDS) efforts in the context of reef-fringed beaches. It is worth noting that most published coastal survey data are concentrated around expansive open coastlines or broad embayed beaches. This dataset introduces an additional resource to benchmark satellite derived shoreline at reef-fringed beaches.

If these data are used for SDS benchmarking efforts, we suggest using one of the produced shoreline contours (LWM, MSL, or MHHW). In reference to MSL (0 m) for Honolulu Tide Gauge, MHHW is at an elevation of 0.329 m. The LWM represents the top of the toe and can be interpreted as a contour of the Mean Lower Low Water (−0.251 m relative to MSL). It is worth noting that the MSL and MHHW contour may contain higher uncertainties compared to the manually collected LWM contour due to the interpolation applied across the beach foreshore.

Other relevant environment data when exploring these data include water level obtained from the Honolulu tide gauge^45^, accessible through the University of Hawaii Sea Level Center with station ID of UHSLC ID 57 (https://uhslc.soest.hawaii.edu/stations/?stn=057#levels). You can access a processed dataset of nearshore wave information, generated using the Simulated WAves Nearshore (SWAN) model. This dataset provides data at a 500-meter grid resolution covering the period from June 20, 2010, to present from PacIOOS (https://www.pacioos.hawaii.edu/metadata/swan_oahu.htm) via THREDDS or OPENDAP. Additionally, data from a wave buoy located on the south shore of O‘ahu near the entrance to Pearl Harbor is available from PacIOOS (https://www.pacioos.hawaii.edu/waves/buoy-pearl/). However, waves originating from the east, which typically wrap around the southeastern corner of O‘ahu, are not always captured by this buoy. For users interested in elevation data beyond the beach, publicly accessible coastal DEMs are available through NOAA Digital Coast (https://coast.noaa.gov/digitalcoast/data/coastallidar.html). We recommend using the 2013 Topo-Bathy LiDAR DEM as of the date of this publication.

## Data Availability

There is no custom code for this project.
